# Time to reflect on diversity, equity, and inclusion in otolaryngology head and neck surgery academic setting

**DOI:** 10.1016/j.bjorl.2023.101319

**Published:** 2023-09-01

**Authors:** Carlos Takahiro Chone

**Affiliations:** Department Otolaryngology-Head and Neck Surgery, University of Campinas, Sao Paulo, Brazil

The diversity among the Brazilian population in racial and ethnic miscegenation is one of the highest in the world. Although diversity, equity, and inclusion have become increasingly focused recently, with the nonwhite population increasing in the United States and women representing more than half of the people, accounting for rising matriculants in medical schools, underrepresented minorities (URM) in academic faculty are only 8% and women 37%.^1^ In our specialty, underrepresented minorities and women are only 2% and 31% in academic otolaryngologist departments, respectively. A recent report from the Association of American Medical Colleges on the rate of full-time medical school faculty among the otolaryngology departments shows that only 10.5% of positions are for women.^2^ Otolaryngology-Head and Neck Surgery was one of five residency specialties with the least female representation (36%).^3^ The specialty is ranked amongst the lower ethnical and racial diversity. Black medical students reported being discouraged from pursuing otolaryngology due to a lack of mentorship and mentors of the same race. Increasing the diversity in the medical workforce and academic medical setting could decrease healthcare inequity in the population.^4^ The Association of American Medical Colleges (AAMC) stated that increasing the diversity of academic health centers is a vital strategy to improve healthcare access in the United States.^5^ John Hopkins Department of Otolaryngology-Head and Neck Surgery formally adopted a diversity inclusion environment in 2004. After ten years, the rate of clinical faculty professors as women went from 5.8% to 23.7%. Improving the diversity in an academic medical faculty could bring innovation and reduction in healthcare disparities.^1^ Diversity brings new lights to thought with improved troubleshooting abilities and innovation, pursuing the entire community to new capabilities and thinking methods already observed in scientific data. Academic and hospital settings should also represent the diversity of the population with an increasing rate of women and URM improving the experience of care by the population.^6^ There is also a great move toward diversity, equity, and inclusion in Brazil, including the officially-engaged post-graduation programs. Still, a lack of scientific data in our field in Brazil left us clueless as the rate of disparities could be the same as in the United States; an inclusive program in academic faculties could shed new light on healthcare pathway improvement.

## Conflict of interest

The authors declare no conflicts of interest.
